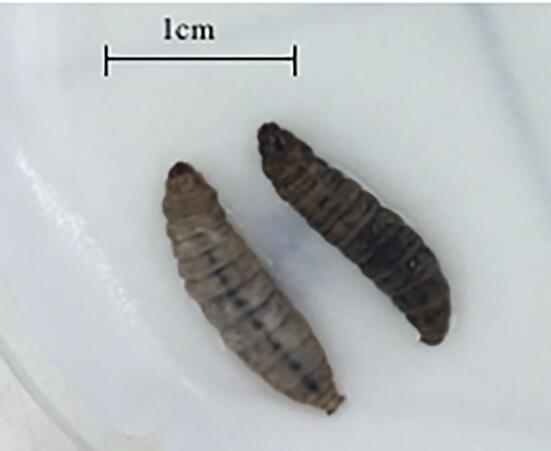# Cordycepin, a metabolite of *Cordyceps militaris,* reduces immune-related gene expression in insects

**DOI:** 10.1016/j.jip.2020.107480

**Published:** 2020-11

**Authors:** Victoria C. Woolley, Graham R. Teakle, Gillian Prince, Cornelia H. de Moor, David Chandler

**Affiliations:** a Warwick Crop Centre, School of Life Sciences, University of Warwick, Wellesbourne, Warwick CV35 9EF, UK; b School of Pharmacy, University of Nottingham, University Park, Nottingham NG7 2RD, UK

**Keywords:** Cordycepin, *Cordyceps militaris*, Secondary metabolite, Entomopathogenic fungi, Insect immunity, *Galleria mellonella*

## Abstract

Hypocrealean entomopathogenic fungi (EPF) (Sordariomycetes, Ascomycota) are natural regulators of insect populations in terrestrial environments. Their obligately-killing life-cycle means that there is likely to be strong selection pressure for traits that allow them to evade the effects of the host immune system. In this study, we quantified the effects of cordycepin (3′-deoxyadenosine), a secondary metabolite produced by *Cordyceps militaris* (Hypocreales, Cordycipitaceae), on insect susceptibility to EPF infection and on insect immune gene expression. Application of the immune stimulant curdlan (20 µg ml^−1^, linear beta-1,3-glucan, a constituent of fungal cell walls) to *Drosophila melanogaster* S2r+ cells resulted in a significant increase in the expression of the immune effector gene *metchnikowin* compared to a DMSO-only control, but there was no significant increase when curdlan was co-applied with 25 µg ml^−1^ cordycepin dissolved in DMSO. Injection of cordycepin into larvae of *Galleria mellonella* (Lepidoptera: Pyralidae) resulted in dose-dependent mortality (LC_50_ of cordycepin = 2.1 mg per insect 6 days after treatment). Incubating conidia of *C. militaris* and *Beauveria bassiana* (Hypocreales, Cordycipitaceae; an EPF that does not synthesize cordycepin) with 3.0 mg ml^−1^ cordycepin had no effect on the numbers of conidia germinating *in vitro*. Co-injection of *G. mellonella* with a low concentration of cordycepin (3.0 mg ml^−1^) plus 10 or 100 conidia per insect of *C. militaris* or *B. bassiana* caused a significant decrease in insect median survival time compared to injection with the EPF on their own. Analysis of predicted vs. observed mortalities indicated a synergistic interaction between cordycepin and the EPF. The injection of *C. militaris* and *B. bassiana* into *G. mellonella* resulted in increased expression of the insect immune effector genes *lysozyme*, *IMPI* and *gallerimycin* at 72 h post injection, but this did not occur when the EPF were co-injected with 3.0 mg ml^−1^ cordycepin. In addition, we observed increased expression of *IMPI* and *lysozyme* at 48 h after injection with *C. militaris*, *B. bassiana* and sham injection (indicating a wounding response), but this was also prevented by application of cordycepin. These results suggest that cordycepin has potential to act as a suppressor of the immune response during fungal infection of insect hosts.

## Highlights

• High doses of cordycepin are lethal to *G. mellonella*.
• Cordycepin interacts with EPF to increase the rate of *G. mellonella* mortality.
• Cordycepin reduces immune-related gene expression in *G. mellonella* and *S2r+* cells.

## Graphical abstract

**Figure:**
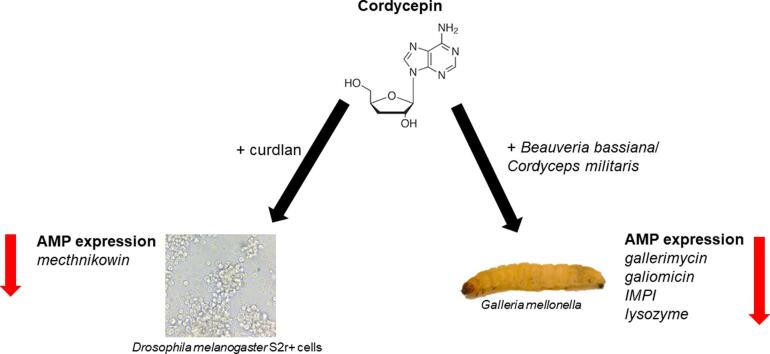

## 1 Introduction

In order to successfully grow and reproduce in its host, a pathogen must evade the effects of the host immune system. This can be done through passive mechanisms (e.g. avoiding immune detection) or by actively interfering with host immune responses (Schmid-Hempel, 2009). Understanding the strategies used by pathogens for immune evasion provides valuable insights into the evolution of virulence in host-parasite relationships (Schmid-Hempel, 2009) but our present understanding of immune evasion in insect pathogens is not well developed.

Hypocrealean entomopathogenic fungi (EPF) (Sordariomycetes, Ascomycota) are obligately-killing parasites that infect a wide range of insects and are common in terrestrial ecosystems (Vega et al., 2009). Infection occurs by percutaneous growth of fungal spores into the haemocoel, from where the fungus starts to proliferate and consume host tissues, resulting in insect death (Chandler, 2016). In laboratory assays insect death usually occurs 3–7 days after infection, however this can take longer in a natural situation and varies between EPF species (Chandler, 2016). If environmental conditions are favourable, the fungus grows back out of the insect cadaver to produce ascospores from fruiting bodies (for sexually reproducing forms) or vegetative conidia (for asexual reproduction), which are released into the environment to be acquired by naïve hosts. This particular life cycle, with transmission being dependent upon the pathogen killing its host, imposes a strong selection pressure for traits that confer high virulence in order to maximise pathogen fitness (Roy et al., 2006, Boomsma et al., 2014). Hypocrealean EPF produce a range of secondary metabolites during infectious growth (de Bekker et al., 2013), and there is experimental evidence that some of these disrupt cellular and systemic immune defences, such as the destruxins, a group of cyclic hexadepsipeptides produced by *Metarhizium* species (Vilcinskas et al., 1997a, Vilcinskas et al., 1997b, Pal et al., 2007, Zimmermann, 2007a, Zimmermann, 2007b). Indirect evidence on the anti-immune function of some other EPF metabolites comes from their use as immune suppressors in human medicine. Examples include cyclosporine, which is used to treat autoimmune diseases and to prevent rejection in organ transplantation, and which is a natural product from *Tolypocladium inflatum* (Hypocreales, Ophiocordycipitaceae) (Survase et al., 2011), as well as the immune-modulating drug fingolimod, used in the treatment of multiple sclerosis, and which is a synthetic derivative of myriocin, produced by *Cordyceps*/*Isaria cicadae* s.l. (previously known as *Isaria sinclairii*, currently undergoing taxonomic revision; Hypocreales, Clavicipitaceae) (Fujita et al., 1994, Strader et al., 2011, Kepler et al., 2017). The homologies between the innate immune systems of mammals and insects (Stokes et al., 2015) probably explains why immunosuppressing metabolites from EPF are also able to affect the human immune system. The innate immune systems are sufficiently similar that insects are also being used increasingly as *in vivo* models of pathogen virulence in medical research (Sheehan et al., 2018).

Here, we present the results of a study on insect immune interference by cordycepin (3′-deoxyadensoine), a secondary metabolite produced by the teleomorphic hypocrealean EPF *Cordyceps militaris* (Hypocreales, Cordycipitaceae) (Cunningham et al., 1950, Shih et al., 2007, Tuli et al., 2014). This fungus occurs naturally in temperate and sub-tropical areas within Asia, Europe, and North and South America, although it is considered to be rare (Shrestha et al., 2012). It is a host specialist, causing natural infections predominantly in lepidopteran larvae and pupae (Shrestha et al., 2012). Cordycepin has been investigated previously for its pharmacological potential, particularly in connection with the use of *C. militaris* fruiting bodies as a traditional herbal medicine (Paterson, 2008). It has been reported to have anti-inflammatory (Kim et al., 2006, Jeong et al., 2010, Kondrashov et al., 2012, Zhang et al., 2014, Yang et al., 2015, Ashraf et al., 2019) as well as anti-tumour (Nakamura et al., 2006) and anti-angiogenic properties (Lu et al., 2014) in studies with mammalian cells, but little is known about its effect on insects. For the present study, we addressed the topic using *Drosophila melanogaster* (Diptera: Drosophilidae) cell culture and *Galleria mellonella* (Lepidoptera: Pyralidae) live caterpillars as experimental systems (Lemaitre et al., 1995, Mylonakis et al., 2005, Bergin et al., 2006, O’Neill, 2006, Ramarao et al., 2012, Harding et al., 2013). Firstly, we quantified the effect of cordycepin on the expression of immunity-related genes in a *D. melanogaster* S2r+ cell line treated with activators of the immune response. Secondly, we measured the survival of *G. mellonella* larvae, together with the expression of immunity-related genes, following injection with cordycepin on its own and in combination with *C. militaris* and *Beauveria bassiana* (Hypocreales, Cordycipitaceae), a related, asexually-reproducing EPF species that does not synthesize cordycepin (Xia et al., 2017). The aim was to provide new information on the effect of cordycepin on insect immune gene expression under the controlled conditions achievable using a cell-based assay, as well as the more biologically-complex conditions of a whole-animal environment.

## 2 Materials and methods

### 2.1 Experimental material

*D. melanogaster* S2r+ cells (Schneider, 1972, Rämet et al., 2001) were cultured in 15 ml cell culture flasks (VWR, Radnor, USA) with 12 ml of Schneider Drosophila Insect Medium (Thermo Fisher Scientific, Waltham, USA) supplemented with 10% foetal bovine serum (FBS; Sigma-Aldrich) and 10% penicillin-streptomycin (Sigma-Aldrich, St Louis, USA). Cell density was determined by trypan blue staining (Strober, 2001). Cells were subcultured when their density reached 1 × 10^7^ cells ml^−1^. All cells used in experiments were between passages 5–15.

Final instar, non-diapausing *G. mellonella* larvae were purchased from Wazp Brand UK Ltd. (Yorkshire, UK). They were maintained at 15 °C in darkness and larvae between 200 and 300 mg were selected for experiments. Cordycepin (Sigma-Aldrich, St Louis, USA) was prepared as a stock solution (100 mg ml^−1^ in DMSO; Thermo Fisher Scientific, Waltham, USA) and diluted with sterile 0.01% Triton X-100 (Merck, Darmstadt, Germany) wetting agent in DEPC-treated water plus 0.1 mg ml^−1^ pentostatin (2′-deoxycoformycin; Sigma-Aldrich, St Louis, USA) (Johnston, 2011).

Laboratory bioassays with entomopathogenic fungi were done using *B. bassiana* strain 433.99 and *C. militaris* strain ARSEF 11703 (Supplementary Table 1). Stock cultures of the strains were stored in cryopreservation (Chandler, 1994). Laboratory cultures were grown from stock cultures on Sabouraud Dextrose Agar (SDA; Thermo Fisher Scientific, Waltham, USA) slopes and maintained at 5 °C for up to six months. Subcultures for laboratory experiments were grown on SDA from slope cultures and incubated in darkness at 23 °C for 10–14 days. Conidia were harvested in sterile 0.01% Triton X-100 and suspensions filtered through milk filters (Lantor Ltd, Bolton, UK) to remove hyphal fragments. Conidia were counted using an Improved Neubauer haemocytometer and aliquots prepared at different concentrations as required.

### 2.2 Effect of cordycepin treatment on the response of *D. melanogaster* S2r+ cells to simulated immune challenge

*D. melanogaster* S2r+ cells were cultured as described above. Cells were collected by centrifugation at 1500 × g for 5 min at 4 °C, the supernatant removed and cells resuspended in Schneider Drosophila Insect Medium with 10% FBS. Aliquots (3 ml) were pipetted into each well of a 6-well plate (TPP tissue culture plate, Sigma-Aldrich, St Louis, USA) at a density of approximately 8 × 10^5^ cells ml^−1^. To determine the effect of cordycepin on gene expression, cells were treated with the immune response stimulants curdlan (linear beta-1,3-glucan; 20 µg ml^−1^; Carbosynth Ltd., Compton, UK) or crude LPS (lipopolysaccharide; 20 µg ml^−1^; Sigma-Aldrich, St Louis, USA), followed by addition of 25 µg ml^−1^ (100 μM) cordycepin solution in DMSO, or DMSO only. An untreated control was also included. Curdlan is structurally similar to fungal cell wall polysaccharides and is used to stimulate an anti-fungal immune response (Kumar et al., 2009), whereas crude LPS (which also contains peptidoglycan) is found in the outer membrane of gram-negative bacteria and is used to stimulate an anti-(gram-negative) bacterial immune response (Tanji and Ip, 2005). Cells were incubated for 4 h at 25 °C, then RNA was extracted ahead of RT-qPCR (see below).

### 2.3 Effect of cordycepin treatment on the survival of *G. mellonella* larvae infected with the EPF *C. militaris* and *B. bassiana*

#### 2.3.1 Dose-response bioassays

Dose response bioassays were done with cordycepin and EPF alone against *G. mellonella* larvae. For the cordycepin dose-response bioassay, 10 final instar *G. mellonella* larvae were cooled on ice for 5 min, then injected in the right front proleg with 30 μl cordycepin at 1.0, 1.8, 3.3, 6.0 or 11 mg ml^−1^ (equivalent to 30, 55, 100, 180, and 330 μg per insect respectively, with a sham injection control) using a 0.3 ml microfine insulin syringe (BD, Franklin Lakes, USA). Where the suspension bled from larvae following injection, the larvae were discarded and not included in the experiment. Immediately after treatment, insects were placed individually in Petri dishes on damp filter paper, sealed with Parafilm, and maintained in darkness at 20 °C. Survival of the larvae was monitored every 24 h for seven days. The bioassay was repeated on three separate occasions, resulting in a total of 30 larvae in each treatment. The DRC package in R (Ritz et al., 2015) was used to estimate lethal concentration (LC) and lethal dose (LD) values at day 6. The EPF dose response bioassay was done in the same way, with final instar *G. mellonella* larvae being injected with 30 μl of conidia suspensions of *C. militaris* and *B. bassiana* at concentrations of 1 × 10^3^, 1 × 10^4^, 1 × 10^5^, 1 × 10^6^ and 1 × 10^7^ conidia ml^−1^ (equivalent to 30, 300, 3 × 10^3^, 3 × 10^4^, 3 × 10^5^ conidia per insect respectively) in sterile 0.01% Triton X-100 (10 insects per treatment, sham injection controls, three independent repeats, LC and LD estimations at day 6).

#### 2.3.2 EPF spore germination

The effect of cordycepin on EPF spore germination was measured as follows: for both *B. bassiana* and *C. militaris*, 100 μl conidia suspension (1 × 10^7^ conidia ml^−1^ in sterile 0.01% Triton X-100) was combined with 30 μl of cordycepin (100 mg ml^−1^) and made up to a final volume of 1 ml using Sabouraud Dextrose Broth (Sigma-Aldrich, St Louis, USA). This gave final concentrations of 1 × 10^6^ conidia ml^−1^ and 3.0 mg ml^−1^ (12 mM) cordycepin (equivalent to the LC_15_ in the *G. mellonella* dose response bioassay). The mixture was incubated in darkness at 23 °C for 24 h, after which the germination of approximately 100 conidia was recorded by examination under a microscope. A conidium was considered germinated if the germ tube was longer than the length of the conidium. The experiment was performed on three occasions. Percentage conidia germination was analysed using a one-way ANOVA following a logit transformation (Warton and Hui, 2011) and statistical normality testing (Shapiro and Wilk, 1965) in SPSS (IBM Corp., 2016).

#### 2.3.3 Effect of cordycepin application on *G. mellonella* susceptibility to EPF

A bioassay was done to measure insect survival following EPF infection supplemented with cordycepin. Batches of 20 final instar *G. mellonella* larvae were injected, as described above, with 30 μl conidia suspension of *B. bassiana* or *C. militaris* at concentrations of 3.3 × 10^2^ and 3.3 × 10^3^ conidia ml^−1^ (equivalent to 10 and 100 conidia per insect respectively) in 0.01% Triton X-100 with/without 3.0 mg ml^−1^ cordycepin. Where the suspension bled from larvae following injection, the larvae were discarded and not included in the experiment. Assessment of larval survival was done as described above for 11 days and the experiment was repeated on three separate occasions, resulting in a total of 30 larvae for each treatment. For each insect batch, 10 individuals were used for survival analysis, while 5 individuals were removed at 0 h (t_0_), 48 h and 72 h, and snap frozen in liquid nitrogen for RNA extraction and quantification of gene expression (Section 2.4). Changes in insect survival were visualised using a Kaplan-Meier estimator, and analysed using a Cox proportional-hazards regression model with replicate and treatment as factors, in which the median survival time (MST) of the insect populations of each treatment and their 95% confidence intervals were calculated, and pairwise comparisons were done using a log-rank χ^2^ test (Cox, 1972; IBM SPSS Statistics Version 24; Bewick et al., 2004). The outcome of combining cordycepin with *B. bassiana* or *C. militaris* on total percentage mortality (synergism, antagonism, or additive effect) at day 6 and day 11 was investigated using the fractional product method for combination treatments, where the effect of the combination is given as (1 − X), where X  = (1 − A)(1 − B) and where A = the proportional effect of agent A on its own, and B is the proportional effect of agent B (Webb, 1963).

### 2.4 Quantification of expression of insect immune-associated genes

RNA was extracted from *D. melanogaster* S2r+ cells using the ReliaPrep RNA Cell Miniprep system (Promega, Madison, USA) following manufacturer’s instructions. For RNA extraction from *G. mellonella*, individual snap frozen larvae were ground in liquid nitrogen using an autoclaved mortar and pestle. RNA was extracted from 50 mg of material using the phenol: chloroform method with Tri-reagent (Sigma-Aldrich, St Louis, USA) then treated with DNase I (Sigma-Aldrich, St Louis, USA) following manufacturer’s instructions. After washing with 75% ethanol, air dried RNA pellets were resuspended in 50 μl DEPC-treated water and stored at −80 °C. RNA concentrations were measured using a NanoDrop ® ND-100 spectrophotometer (Thermo Fisher Scientific, Waltham, USA). cDNA was synthesized using Superscript kits (Thermo Fisher Scientific, Waltham, USA): Superscript III was used to reverse transcribe *D. melanogaster* RNA (100 μg) and Superscript II was used for *G. mellonella* RNA (500 μg), both with random hexamers. Manufacturer’s instructions were followed, with the exception that 0.5 μl (100 units) SuperScript III was used for reactions. The expression of insect immune associated genes was quantified by RT-qPCR using a Lightcycler 480 (Roche Holding AG, Basel, Switzerland). For *D. melanogaster* S2r+ cells, expression levels were quantified for genes encoding the ribosomal protein RP49 (used as the reference), and the antimicrobial peptides (AMPs) metchnikowin and attacin A. For *G. mellonella*, expression levels were quantified for genes encoding the following proteins: the ribosomal protein S7e (reference); the AMPs gallerimycin and galiomicin; lysozyme; and the insect metalloproteinase inhibitor (IMPI). Gene expression in *G. mellonella* was measured for larvae injected with EPF +/− exogenous cordycepin, as well as for sham injections and uninjected controls. Primers for all genes are shown in Table 1. SensiFAST SYBR No-ROX (Bioline, London, UK) was used for RT-qPCR, each reaction contained: 5 μl SYBR Green, 2 μl DEPC treated water, 1 μl forward primer (10 μM), 1 μl reverse primer (10 μM) and 1 μl cDNA. The conditions were: 95 °C 2 min, 40× (95 °C 5 s, 60 °C 10 s and 72 °C 20 s). The comparative *C_T_* method (also known as the $2^{-\Delta \Delta CT}$ method) (Schmittgen and Livak, 2008) was used to calculate fold changes in gene expression compared to untreated samples with correction for the internal control. Each gene of interest was analysed independently. For *D. melanogaster*, data on relative gene expression were tested for normality (Shapiro and Wilk, 1965) before being subject to ANOVA, while for *G. mellonella*, the data were tested for normality and then analysed using Kruskal-Wallis H tests (IBM SPSS Statistics Version 24). Where overall significant differences were observed, post-hoc analysis was performed using a Tukey HSD post hoc test (following ANOVA) or the Dunn-Bonferroni post hoc method (following Kruskal-Wallis tests).

**Table 1:** Primers used to monitor immune-related gene expression in *G. mellonella* and *D. melanogaster*.

Gene	Primer sequence (5′-3′)	Reference
*gallerimycin*	TATCATTGGCCTTCTTGGCTG	Wojda and Jakubowicz (2007)
	GCACTCGTAAAATACACATCCGG	
*galiomicin*	TCGTATCGTCACCGCAAAATG	Wojda et al. (2009)
	GCCGCAATGACCACCTTTATA	
*lysozyme*	TCCCAACTCTTGACCGACGA	Altincicek and Vilcinskas (2006)
	AGTGGTTGCGCCATCCATAC	
*IMPI*	AGATGGCTATGCAAGGGATG	Altincicek and Vilcinskas (2006)
	AGGACCTGTGCAGCATTTCT	
*S7e*	ATGTGCCAATGCCCAAGTTG	Wojda and Jakubowicz (2007)
	GTGGCTAGGCTTGGGAAGAAT	
*attacin A*	AGGTTCCTTAACCTCCAATC	Jin et al. (2008)
	CATGACCAGCATTGTTGTAG	
*metchnikowin*	TCTTGGAGCGATTTTTCTGG	Castillo et al. (2013)
	AATAAATTGGACCCGGTCTTG	
*RP49*	GACGCTTCAAGGGACAGTATCTG	Gobert et al. (2003)
	AAACGCGGTTCTGCATGAG	

## 3 Results

### 3.1 Effect of cordycepin treatment on gene expression in *D. melanogaster* S2r+ cells following immune challenge

**Fig. 1:**
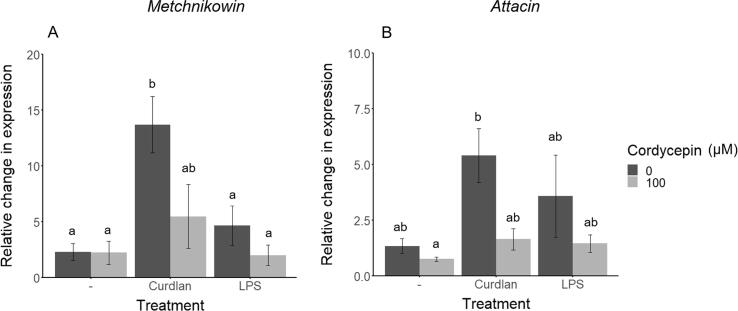
Expression of AMPs in *D. melanogaster* S2r+ cells in response to cordycepin treatment. S2r+ cells (three biological replicates) were treated with DMSO (dark grey bars) or DMSO plus 25 µg ml^−1^ (100 µM) cordycepin (light grey bars). Immune challenge was simulated using 20 µg ml^−1^ LPS or curdlan. Cells were sampled 4 h after treatment. Fold changes in gene expression were calculated using the $2^{-\Delta \Delta CT}$ method (Schmittgen and Livak, 2008) compared to untreated samples with correction for the internal control (RP49). Relative expression of A) *metchnikowin* and B) *attacin* A is shown. Error bars show ± SEM and different lowercase letters indicate significant differences (p < 0.05; ANOVA, Tukey HSD post hoc tests).

The relative expression of the immune effector genes *metchnikowin* and *attacin* was quantified in a *D. melanogaster* S2r+ cell line treated separately with two activators of the immune response, curdlan and LPS, with and without the addition of 25 µg ml^−1^ cordycepin (Fig. 1). There was significant variation among treatments in the relative expression for both *metchnikowin* (*F_5,17_* = 5.93, P < 0.01) and *attacin* A (*F_5,17_* = 3.48, P < 0.05). Application of curdlan significantly elevated the mean relative expression of *metchnikowin* compared to the DMSO-only control or DMSO plus cordycepin (Tukey HSD, P < 0.05, approximately 6-fold increase). When cordycepin was co-applied with curdlan, there was no significant difference in mean relative gene expression compared to DMSO-only or DMSO plus cordycepin (P > 0.05, approximately 2.5-fold change), nor was there a significant difference between the mean relative expression of *metchnikowin* for the curdlan-only treatment vs. curdlan + cordycepin (P > 0.05, 2.5-fold change). Treatment with LPS, or LPS + cordycepin, did not have a significant effect on relative expression of *metchnikowin* compared to the DMSO-only control (P > 0.05, 2-fold increase for LPS only, no fold change for LPS + cordycepin) (Fig. 1A). For *attacin A*, post hoc comparisons showed that relative expression was significantly higher following treatment with curdlan compared to the DMSO plus cordycepin- treatment (P < 0.05, approximately 7-fold increase) (Fig. 1B).

### 3.2 Effect of cordycepin treatment on the survival of *G. mellonella* larvae infected with *C. militaris* and *B. bassiana*

In this set of experiments we investigated whether application of exogenous cordycepin would affect the susceptibility of *G. mellonella* larvae to fungal infection and impact on the expression of host immune genes. We used a low dose of cordycepin that did not inhibit fungal activity and did not cause excessive insect mortality on its own.

#### 3.2.1 Dose-response bioassays of *B. bassiana* and *C. militaris* against *G. mellonella* larvae

**Fig. 2:**
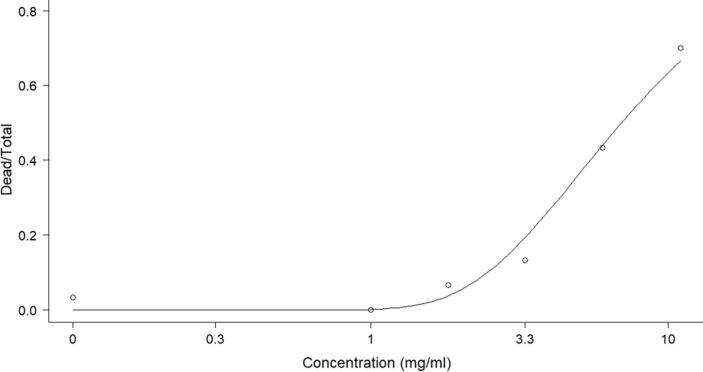
Proportion mortality of *G. mellonella* larvae following cordycepin treatment. Mean proportional mortality of *G. mellonella* larvae (N = 30 for each dose) injected with 1, 1.82, 3, 6 and 11 mg ml^−1^ of cordycepin and a DMSO control on day 6 after treatment. A type 1 Weibull model is fitted to the response.

**Fig. 3:**
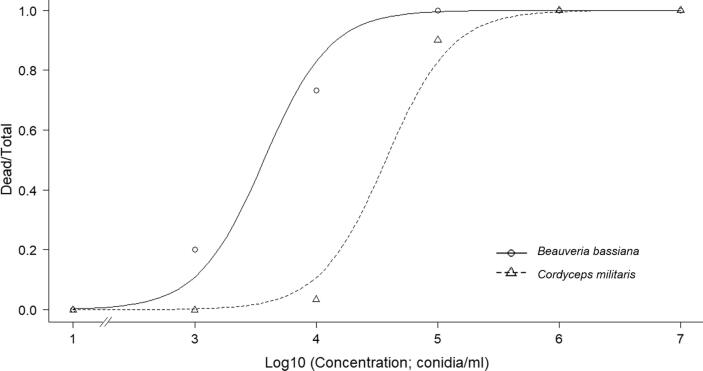
Proportion mortality of *G. mellonella* larvae following treatment with EPF. Mean proportional mortality of *G. mellonella* larvae (N = 30 for each dose) injected with *B. bassiana* and *C. militaris* conidia at six concentrations on day 6 after treatment. A two-parameter log-logistic model is fitted to the responses.

Larvae of *G. mellonella* were treated with either EPF or cordycepin to established the impact of these treatments individually on insect survival. There was a positive relationship between *G. mellonella* mortality and dose of cordycepin applied (Fig. 2, Weibull type 1 model), the LC_15_, LC_50_, LD_15_ and LD_50_ were also calculated on day 6 after treatment and found to be 2.91 (±0.35) mg ml^−1^, 6.96 (±1.03) mg ml^−1^, 87.95 (±10.79) µg per larvae and 209.34 (±30.70) µg per larvae, respectively. Individual larvae receiving a lethal dose of cordycepin became distinctly grey in colour between 24 and 48 h prior to death (Supplementary Fig. 1) and their movement declined markedly. After death, cadavers turned black and their abdomens were fluid-filled. *G. mellonella* larvae were susceptible to lethal infections by both *B. bassiana* and *C. militaris* (Fig. 3; estimated *B. bassiana* LC_50_ (day 6) = 3.6 × 10^3^ (±9.4 × 10^2^) conidia ml^−1^, LD_50_ = 1.1 × 10^2^ (±2.8 × 10^1^) conidia per insect; *C. militaris* LC_50_ = 4.0 × 10^4^ (±9.1 × 10^3^) conidia ml^−1^, LD_50_ = 1.2 × 10^3^ (±2.8 × 10^2^) conidia per insect; two-parameter log-logistic model).

#### 3.2.2 EPF conidia germination

**Fig. 4:**
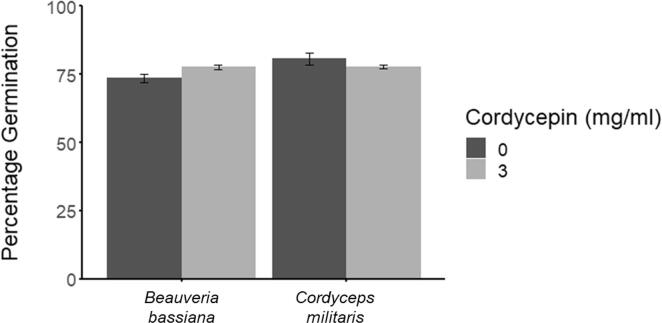
The impact of cordycepin on EPF germination. Mean (back-transformed) percentage germination of *B. bassiana* 433.99, *C. militaris* 11703, and *M. brunneum* 275.86 conidia ± SE following incubation for 24 h with 0 (dark grey) and 3 (light grey) mg ml^−1^ of cordycepin.

Conidia of *C. militaris* and *B. bassiana* were incubated with cordycepin to identify any inhibitory effects on germination. There was no significant effect of cordycepin treatment (3 mg ml^−1^) on the numbers of *B. bassiana* and *C. militaris* conidia germinating *in vitro* after 24 h (F_3,11_ = 1.045, P > 0.05). The mean germination (back-transformed) of *B. bassiana* conidia populations was 75% and 79% at 0 and 3 mg ml^−1^ of cordycepin respectively, while for *C. militaris* conidia it was 82% and 79% respectively (Fig. 4).

#### 3.2.3 Effect of cordycepin application on *G. mellonella* susceptibility to fungal infection

**Fig. 5:**
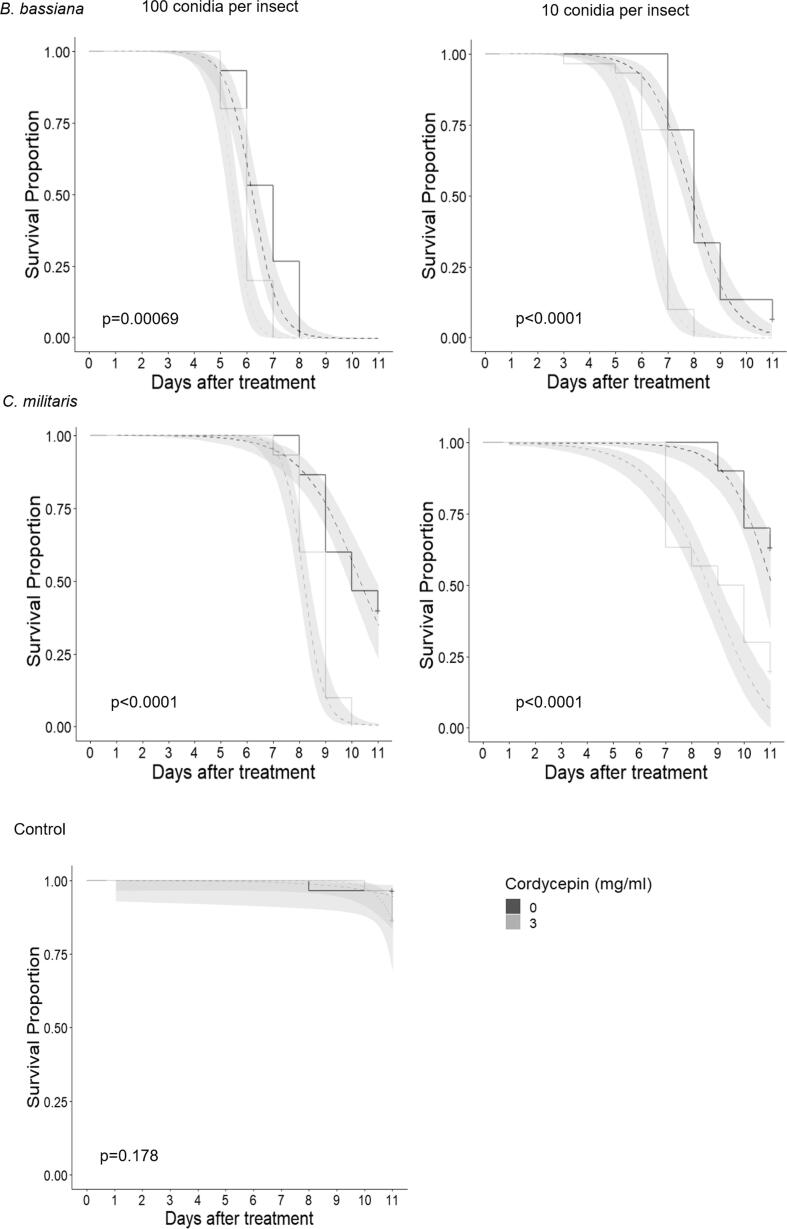
Survival curves of *G. mellonella* treated with cordycepin and EPF. Survival of *G. mellonella* larvae injected with 10 or 100 *B. bassiana* conidia or *C. militaris* conidia. Solid lines show observed mortalities, those lines ending in ‘+’ indicate censored populations. Dashed lines indicate the expected population decline (shaded area illustrates the standard error) estimated by fitting a binomial generalized logistic model. Pale grey lines indicate treatment with cordycepin (3 mg ml^−1^) and dark grey with a DMSO control. N = 30 per treatment. P values are the result of a log-rank test.

A laboratory bioassay was done to quantify the effects on *G. mellonella* survival of co-applying 3 mg ml^−1^ cordycepin with *C. militaris* or *B. bassiana* at doses of 10 and 100 conidia per insect. Mortality of *G. mellonella* larvae in the sham injection control was 3.3% at 11 days post injection (dpi) (Supplementary Table 2). Injection of larvae with 3 mg ml^−1^ cordycepin resulted in a mortality of 13.3%, which was in keeping with the results from the earlier cordycepin dose response bioassay (Fig. 2) but was not statistically significantly different from the sham injection control (Supplementary Table 2). Compared to injection of EPF on their own, co-injection of 3 mg ml^−1^ cordycepin with *B. bassiana* or *C. militaris* caused a significant decrease in median survival time of c. 24 h in all cases (Supplementary Table 2, log rank chi squared > 3.841, P < 0.05; also see survival curves, Fig. 5) and this was reflected by an increase in the hazards ratio (Supplementary Table 2). A dose of 10 conidia per insect of *C. militaris* resulted in 36.7% mortality at 11 dpi, meaning that median survival time could not be estimated (Supplementary Table 2). At day 6 the predicted mean mortality of *B. bassiana* + cordycepin combination, calculated from the fractional product of *B. bassiana* and cordycepin mortality individually, was 0% at a dose of 10 conidia per insect (observed value = 26.7%) and 46.7% at a dose of 100 conidia per insect (observed value = 80%). At day 11 the predicted mean mortality of the *C. militaris* + cordycepin combination was 45.1% at a dose of 10 conidia per insect (observed value = 80%), and 65.3% at a dose of 100 conidia per insect (observed value = 80%). Both the results on days 6 and 11 after treatment indicate that there was a synergistic effect of the combination treatment with EPF and cordycepin (Webb, 1963).

### 3.3 Effect of cordycepin treatment on the expression of immune-related genes in *G. mellonella* larvae infected with *B. bassiana* and *C. militaris*

**Fig. 6:**
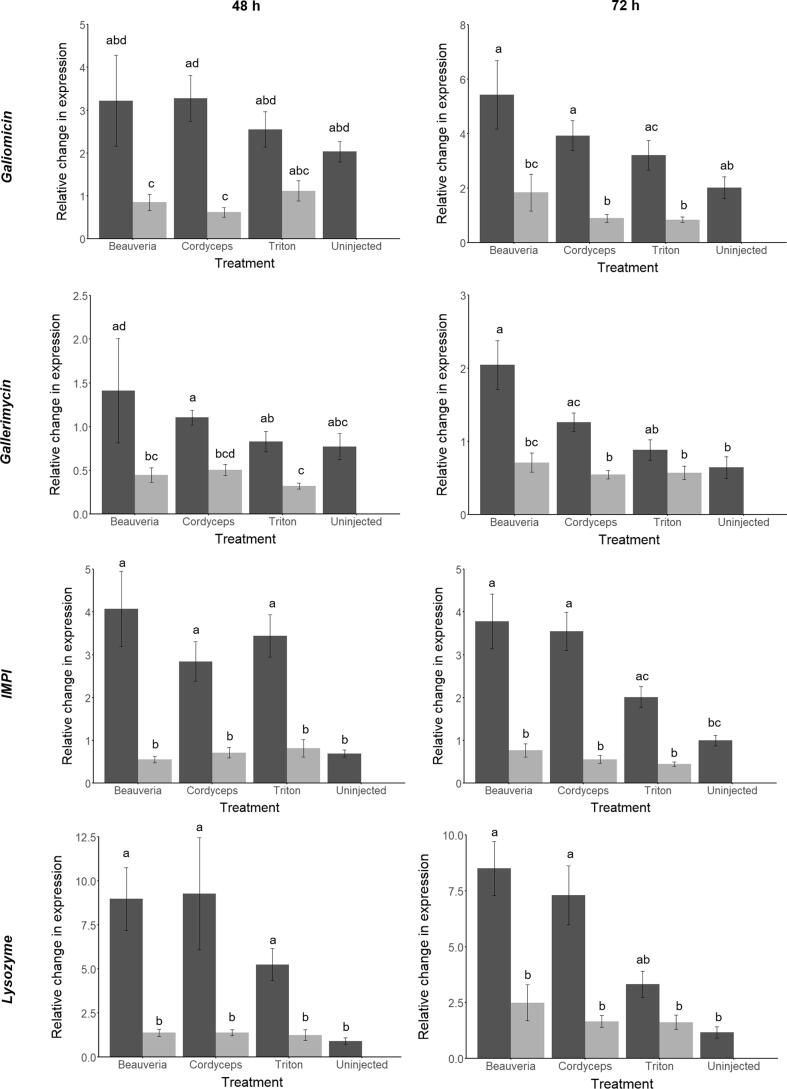
Expression of immune-related genes in response to cordycepin and *C. militaris* or *B. bassiana* 48 h and 72 h after treatment. The expression of galiomicin, gallerimycin, IMPI and lysozyme in response to stimulation by *C. militaris* or *B. bassiana* injection and sham (Triton X-100) injection 48 h and 72 h. Fold changes in gene expression were calculated using the $2^{-\Delta \Delta CT}$ method (Schmittgen and Livak, 2008) compared to untreated samples with correction for the internal control (*S7e*). Treatment without cordycepin is shown by dark grey bars and with cordycepin (3 mg ml^−1^) by light grey bars, expression in uninjected insects is also included. N = 13–15 per treatment. Different letters above bars indicate statistically significant differences (p < 0.05) identified using the Dunn-Bonferroni post hoc method following a Kruskal-Wallis test.

In order to provide information on the possible mechanism by which cordycepin affects fungal infection, the expression of four *G. mellonella* immune-related genes (*galiomicin, gallerimycin*, *IMPI* and *lysozyme*) was quantified at 48 h and 72 h in larvae injected with *B. bassiana* or *C. militaris* +/− cordycepin (Fig. 6). The experiment also included a sham injection control and an untreated (=un-injected) control. Pairwise comparisons (Dunn-Bonferroni method) showed that, at 48 h, injection with *C. militaris*, *B. bassiana*, and sham injection resulted in a significantly larger increase (P < 0.05) in relative expression of *IMPI* and *lysozyme* compared to the untreated control. At 72 h, injection with *C. militaris* and *B. bassiana* resulted in a significantly larger increase (P < 0.05) in relative expression of *gallerimycin*, *IMPI,* and *lysozyme* compared to the untreated control, but there was no significant difference between sham injection and the untreated control for any of the four genes (P > 0.05). In contrast, there was no significant increase (P > 0.05) in relative immune gene expression when cordycepin was co-applied with *C. militaris*, *B. bassiana* or on its own. Addition of cordycepin caused between 2-fold to 7-fold reduction in relative gene expression depending on the EPF species and immune gene (Fig. 6). Compared to injection with *B. bassiana* only, co-injection of *B. bassiana* + cordycepin resulted in a reduction in expression of: (i) *galiomicin* by ~3.5× (P < 0.01) at 48 h and ~3× (P < 0.01) at 72 h; (ii) *gallerimycin* by ~3× at both 48 h (P < 0.05) and 72 h (P < 0.05); (iii) *IMPI* by ~7.5× (P < 0.001) at 48 h and ~5× (P < 0.001) at 72 h; and (iv) *lysozyme* by ~6.5× (P < 0.001) at 48 h and ~3.5× (P < 0001) at 72 h. A similar pattern of reduced gene expression was observed with *C. militaris* and cordycepin. Compared to injection with *C. militaris* only, co-injection of *C. militaris* + cordycepin resulted in a reduction in expression of: (i) *galiomicin* by ~5× (P < 0.001) at 48 h and ~4.5× (P < 0.001) at 72 h; (ii) *gallerimycin* by ~2× at both 48 h (P < 0.01) and 72 h (P < 0.05); (iii) *IMPI* by ~x4 (P < 0.005) at 48 h and ~6× (P < 0.001) at 72 h; and (iv) *lysozyme* by ~6.5× (P < 0.01) at 48 h and ~4× (P < 0.005) at 72 h. Compared to the sham injection control, injection of cordycepin resulted in a reduction in expression of: (i) *galiomicin* by ~3× (P < 0.01) at 72 h; (ii) *gallerimycin* by ~2× (P < 0.02) at 48 h; (iii) *IMPI* by ~3× (P < 0.001) at 48 h and ~3× (P < 0.001) at 72 h; and (iv) *lysozyme* by ~2.5× (P < 0.001) at 48 h.

## 4 Discussion

In order to complete their parasitic life cycle, obligately-killing EPF such as C*. militaris* must nullify the effects of host immune defences (Vey et al., 2002, Wang and Leger, 2006, Pal et al., 2007, Butt et al., 2016). The inference from our findings is that the *C. militaris* secondary metabolite cordycepin is capable of acting as an insect immune modulator, dampening down the upregulation of defence genes in response to fungal infection. This suppression of the immune response may facilitate infection of insects by *C. militaris*. To our knowledge, this is the first report of the effects of cordycepin on immune gene expression in insects.

The statistical variation that we observed in the *D. melanogaster* S2r+ cell assay means that some caution is needed in interpretation of the results, but overall, they suggest that cordycepin may have an inhibitory effect on expression of the anti-fungal AMP gene *metchnikowin*, despite the downregulation in expression of this gene not being significant following curdlan treatment. Specifically, while immune challenge with the anti-fungal elicitor curdlan resulted in increased expression of *metchnikowin* compared to the DMSO-only control, this did not occur when curdlan was applied in the presence of cordycepin, suggesting that cordycepin was able to inhibit upregulation of this immune gene (post hoc comparisons showed no significant difference between treatment with curdlan vs. curdlan + cordycepin, but we expect that higher doses of cordycepin are likely to have resulted in a greater effect on gene expression). Metchnikowin (Levashina et al., 1995), which is specific to members of the Drosophilidae, is synthesized via the Toll and Imd pathways in response to fungal or bacterial infection (De Gregorio et al., 2002, Imler and Bulet, 2005). As expected, treatment with the anti-(gram negative) bacterial elicitor LPS (Tanji and Ip, 2005) did not result in increased *metchnikowin* expression compared to the control. However, unexpectedly, we observed no significant increase in expression of the AMP gene *attacin* A in response to LPS in our study. Attacins are conserved across different species in the Lepidoptera and Diptera, they are broadly considered to be active against gram-negative bacteria (Lemaitre and Hoffmann, 2007, Yi et al., 2014), although in *D. melanogaster* there are multiple *attacin* genes (A, B1, D) which are expressed in response to gram-negative bacteria and fungi. The expression of *attacin* D is controlled by the IMD pathway, which is activated by gram-negative bacteria (De Gregorio et al., 2002). The expression of *attacins* A and B1 is controlled by both the Toll and IMD pathways and responds to infections by fungi and bacteria (De Gregorio et al., 2002). Previous experiments with *Aedes aegypti* (Diptera: Culicidae) and *D. melanogaster* have found that *attacin* is expressed in response to *B. bassiana* infection (Lemaitre et al., 1997, De Gregorio et al., 2002, Dong et al., 2012, Yi et al., 2014). Our finding of significantly increased *attacin A* expression in the *D. melanogaster* S2r+ cell assay in the curdlan vs. cordycepin-only treatment, provides some tentative support that *attacin A* expression is responsive to fungal infection.

The inhibitory effects of cordycepin on the anti-fungal insect immune immune response was further supported by the results from our *G. mellonella* experiment, where application of cordycepin prevented increased expression of different immune effector genes in response to fungal infection, and resulted in faster host insect death. Injection of *G. mellonella* larvae with *B. bassian*a, *C. militaris*, or a sham injection, resulted in increased expression of *lysozyme* and *IMPI* at 48 h after treatment. Meanwhile, at 72 h we observed elevated expression of *lysozyme*, *IMPI*, and *gallerimycin* (but not *galiomycin*) in response to *B. bassiana* and *C. militaris* injection, but there was no elevated immune gene expression in response to sham injection compared to the untreated control, suggesting that the gene expression response to wounding was short lived. Applying exogenous cordycepin alongside *B. bassiana* or *C. militaris*, or as an addition to the sham injection, dampened down the expression of these immune genes so that relative expression was no different from the untreated control. Gallerimycin and galiomycin are both anti-fungal defensins (Schuhmann et al., 2003, Lee et al., 2004, Langen et al., 2006) synthesized via the Toll pathway in response to EPF infection (Vertyporokh and Wojda 2017). Lysozyme has both anti-bacterial and anti-fungal activity (Jollès and Jollès, 1984, Wojda et al., 2009, Sowa-Jasiłek et al., 2016), while IMPI is a *G. mellonella*-specific inhibitor of EPF metalloproteinases that hydrolyses insect proteins in the cuticle and haemolymph (Vilcinskas and Wedde, 2002, Altincicek et al., 2007, Wedde et al., 2007), and is itself a target for EPF proteinases synthesized as a fungal countermeasure to the *G. mellonella* immune response (Mukherjee and Vilcinskas, 2018). Expression of *IMPI* has been shown previously to be up-regulated within 48 h of fungal infection (Vertyporokh and Wojda, 2017). Expression of both *IMPI* and *lysozyme* in *G. mellonella* is reported to be upregulated by a range of stimuli including fungal and bacterial infection (Vertyporokh and Wojda, 2017), metamorphosis and wounding (Griesch et al., 2000, Altincicek and Vilcinskas, 2006, Vilcinskas, 2019). The signalling pathway(s) leading to *IMPI* and *lysozyme* expression in *G. mellonella* have yet to be fully elucidated, although they are likely to be regulated by Toll, Imd or a related pathway as their transcription is controlled by c-Rel proteins Relish, DIF and Dorsal (Altincicek and Vilcinskas, 2006, Vilcinskas, 2019).

We observed no upregulation of *galiomycin* in response to fungal infection in *G. mellonella*, which may have been due to the route of infection (injection rather than topical application) or because gene expression was measured using whole insects rather than specific tissues, such as the fat body, which is a site of AMP synthesis (Vertyporokh and Wojda, 2017). We chose injection as the way to deliver cordycepin to *G. mellonella* as it mimics the normal route of exposure, since host tissues are exposed to it after fungal hyphae have grown into the insect haemocoel. Injection of EPF also allowed us to precisely control the dose of pathogen, and to ensure that cordycepin and EPF were applied at the same time and location.

To date, studies on the effects of cordycepin have focused on the mammalian innate response rather than the insect immune system (for example see Chu and Edelman, 1972, Penman et al., 1970, Kim et al., 2006, Jeong et al., 2010, Kondrashov et al., 2012, Ren et al., 2012, Ashraf et al., 2019). The close similarities between aspects of the mammalian innate immune system and insect immunity - such as the homology between TLR (mammal) and Toll (insect) immune signalling (Sheehan et al., 2018) - would explain why cordycepin is able to affect both mammalian and insect immune responses. Cordycepin treatment is reported to reduce inflammatory mRNAs in primary human airway smooth muscle cells and mouse macrophage cells treated with an inflammatory stimulus (Kondrashov et al., 2012, Ashraf et al., 2019). The precise mechanism(s) of action of cordycepin on mammalian cells is not yet known, although there is evidence that it can prevent nuclear translocation of NFκB in mammalian microglia, embryonic kidney and macrophage cells (Jeong et al., 2010, Ren et al., 2012, Ashraf et al., 2019), while it has also been shown to affect a subset of polyadenylated mRNAs in a variety of human cell types, (Penman et al., 1970, Chu and Edelman, 1972, Kondrashov et al., 2012).

Secondary metabolites play an important role in the pathogenicity of EPF (de Bekker et al., 2013) and - in principle - the deployment of metabolites for active suppression of the insect immune response would be beneficial for fungal fitness. The array of secondary metabolites produced by non-specialist EPF has been shown to vary between species, insect tissue type, and whether fungal growth is infectious or saprotrophic (i.e. in living or dead tissue) (de Bekker et al., 2013). It is possible that some of these compounds function as immunosuppressors, but may also have additional roles. For example, injection of destruxin A into *D. melanogaster*, mimicking infection by *Metarhizium* species, reduced expression of AMPs and made them susceptible to infection by normally non-pathogenic *E. coli* by inhibition of the IMD pathway (Pal et al., 2007). However destruxin A (as well as destruxins B and E) has also been found to have insecticidal activity against *Spodoptera litura* (Lepidoptera) and, in the same study, a crude destruxin preparation was found to have synergistic effects on the pathogenicity of *Cordyceps javanica* (Hypocreales, Cordycipitaceae) (formerly *Paecilomyces javanicus* and *Isaria javanica*) to *S. litura* (Hu et al., 2007), which echoes our finding of a synergistic interaction between co-application of exogenous cordycepin together with *C. militaris* or *B. bassiana*.

Few studies have been done on the mechanisms of pathogenesis by *C. militaris*, but there is evidence that passive avoidance of immune detection is less important for successful infection compared to generalist EPF species (Kryukov et al., 2020). Our finding that cordycepin was able to suppress the expression of different antimicrobial effectors is likely to be of benefit to *C. militaris* as an immune interference strategy, as insect defence compounds are known to operate synergistically against pathogen infections (Kaur et al., 2015, Butt et al., 2016). Therefore, the ability to suppress the activity of a range of defence compounds at once might be a more efficient - and therefore selectively advantageous - strategy than targeting individual compounds with different suppressors. The production of cordycepin by *C. militaris* has been found to vary between 750 and 16500 µg g^−1^ depending on fungal strain, cultivation method and extraction method (Huang et al., 2003, Li et al., 2004, Yang and Li, 2008). Therefore, the amount of cordycepin used in our experiments probably represents a higher dose than that received by a *G. mellonella* larva (which weighed 200–300 mg) when infected with *C. militaris* on its own: the evidence for this is that injection of *C. militaris* into *G. mellonella* still resulted in an increase in expression of immune related genes, even though this fungus is known to synthesize cordycepin. This raises a question of why, if cordycepin is able to supress the immune response and increase the virulence of the fungus, is it not produced at higher levels in natural infections by *C. militaris*? It has been proposed that there is a trade-off between metabolite production and EPF growth, as highly active toxins could result in host mortality before the fungus has grown sufficient biomass to produce structures needed for spore production, and which would also leave the host cadaver vulnerable to fast growing, saprotrophic microbes (Boomsma et al., 2014). In our study, cordycepin was lethal to *G. mellonella* larvae depending on the dose applied. Larvae killed by cordycepin injection exhibited the symptoms of a bacterial infection (Tanada and Kaya, 1993) and we were able to recover opportunist bacterial pathogens from them including *Bacillus* sp., *Enterobacter* sp., *Enterococcus* sp. *Pantoea* sp. and *Serratia* sp. (Woolley, 2018). Similar symptoms were observed by Kim et al., (2002), who found that larvae of the diamondback moth *Plutella xylostella* (Lepidoptera: Plutellidae) fed leaf discs treated with cordycepin (25–500 mg l^−1^) became dark brown and then lysed after death. A methanolic extract of the *C. militaris* fruiting body has been found to exhibit antibacterial and antifungal properties (Reis et al., 2013), which suggests that *in vivo*, *C. militaris* produces metabolites that may prevent opportunistic infections of the fruiting body by bacteria or fungi.

Further work needs to be conducted to elucidate the mode of action of cordycepin during insect infection by *C. militaris*. In particular, it would be important to determine how cordycepin inhibits AMP expression, including effects on the activity of transcription factors in the Toll and Imd pathways (Kondrashov et al., 2012, Ren et al., 2012) and polyadenylation of immune genes (Kondrashov et al., 2012). It would also be worth investigating whether cordycepin has an effect on the cellular immune response, including the prophenol oxidase cascade and wounding response. Although our study suggests that cordycepin may play a role in the virulence of *C. militaris*, conclusive proof would be needed by construction of knock outs of the genes responsible for cordycepin production (Xia et al., 2017). Improved knowledge of the EPF-host immune interaction may help us understand the selection pressures affecting fungal pathogenicity and host immune defences (Boomsma et al., 2014, Mukherjee and Vilcinskas, 2018), as well as practical applications such as the selection and development of EPF strains for biological pest control or the identification of secondary metabolites as novel pesticides, efficacy enhancing agents, or pharmaceuticals (Butt et al., 2016).

## Declaration of Competing Interest

The authors declare that they have no known competing financial interests or personal relationships that could have appeared to influence the work reported in this paper.

## Figures and Tables

**Supplementary figure 1:**